# Investigation and management of resistant hypertension: British and Irish Hypertension Society position statement

**DOI:** 10.1038/s41371-024-00983-6

**Published:** 2024-12-09

**Authors:** Luca Faconti, Jacob George, Sarah Partridge, Carmen Maniero, Abilash Sathyanarayanan, Spoorthy Kulkarni, Vikas Kapil, Alfredo Petrosino, Philip Lewis, Terry McCormack, Neil R. Poulter, Anthony Heagerty, Ian B. Wilkinson

**Affiliations:** 1 Department of Clinical Pharmacology, King’s College London British Heart Foundation Centre, St. Thomas’ Hospital, London, UK; 2 Division of Molecular & Clinical Medicine, School of Medicine, Ninewells Hospital & Medical School, University of Dundee, Dundee, UK; 3 Department of Primary Care and Public Health, Brighton and Sussex Medical School, University of Sussex, Brighton, UK; 4 William Harvey Research Institute, Barts & The London School of Medicine & Dentistry, Queen Mary University of London, London, UK; 5 Nottingham University Hospitals, Nottingham, UK; 6 Division of Experimental Medicine, University of Cambridge, Addenbrooke’s Hospital, Cambridge, UK; 7 Barts Blood Pressure Centre of Excellence, Barts Heart Centre, St Bartholomew’s Hospital, Barts Health NHS Trust, West Smithfield, London, UK; 8 London Tubular Centre, Department of Renal Medicine, University College London, Royal Free Hospital, London, UK; 9 Stockport NHS Foundation Trust, Stockport, UK; 10 Institute of Clinical and Applied Health Research, Hull York Medical School, Hull, UK; 11 Imperial Clinical Trials Unit, School of Public Health, Imperial College London, London, UK; 12 Division of Cardiovascular Sciences, Faculty of Biology, Medicine and Health, University of Manchester, Manchester, UK

**Keywords:** Preventive medicine, Lifestyle modification, Hypertension

## Abstract

People living with resistant hypertension (RH) are at high risk of adverse cardiovascular events. The British and Irish Hypertension Society has identified suspected RH as a condition for which specialist guidance may improve rates of blood pressure control and help clinicians identify those individuals who may benefit from specialist review. In this position statement we provide a practical approach for the investigation and management of adults with RH. We highlight gaps in the current evidence and identify important future research questions. Our aim is to support the delivery of high-quality and consistent care to people living with RH across the UK and Ireland.

## Introduction

Hypertension remains the single most important modifiable risk factor for cardiovascular disease (CVD), disability and death in the world [1, 2]. An estimated 30% of adults in the UK have hypertension, and at least 1/3rd of them fail to achieve the recommended blood pressure (BP) targets despite therapeutic intervention [3]. Such individuals have a 50% greater risk of CVD and kidney disease compared to those who achieve target BP [4]. Although BP measurement errors, lifestyle factors, suboptimal treatment strategies and undiagnosed secondary hypertension may explain poor BP control in some individuals, a proportion of people present with true resistant hypertension (RH) and remain at increased risk of CVD [4–8].

The British and Irish Hypertension Society (BIHS) has identified suspected RH as condition for which specialist guidance may help improve rates of BP control and help clinicians identify those people who may benefit from specialist review. In this statement, we outline the BIHS recommendations for the investigation and management of adults living with RH, based on a critical review of the literature and expert opinion where evidence is lacking. Practical approaches are described to facilitate the delivery of high-quality and consistent care across the UK and Ireland.

## Definition of resistant hypertension

Despite being a common clinical condition there is no universally agreed definition of RH. The National Institute of Clinical Excellence (NICE) defines RH in adults, as BP that remains uncontrolled (i.e., office BP ≥ 140/90 mmHg/out-of-office ≥135/85 mmHg) despite taking optimal tolerated doses of an angiotensin converting enzyme inhibitor (ACE-I) or angiotensin receptor blocker (ARB) in addition to a calcium channel blocker (CCB) and a thiazide-like diuretic [9]. Confirming adherence to antihypertensive agents is also advised. The European Society for Hypertension (ESH) defines RH as clinic BP measurements ≥ 140/90 mmHg, confirmed by out-of-office measurements (24-h ambulatory BP monitor (ABPM) readings ≥130/80 mmHg) despite use of a 3-drug antihypertensive regimen including a diuretic. Evidence of adherence to therapy and exclusion of secondary forms of hypertension are also needed to confirm a diagnosis of RH [10]. The ESH guidelines are endorsed by the International Society of Hypertension [11] and their definition of RH is similar to the one proposed by the European Society of Cardiology (ESC) guidelines for the management of elevated blood pressure and hypertension [12]. The American Heart Association (AHA) [13] and Hypertension Canada [14] guidelines also specify the use of three antihypertensive agents, ‘*commonly including*’ and ‘*preferably including*’ a diuretic respectively. The AHA scientific statement on RH (2018) [15] also includes a separate category of people with BP at target on four or more antihypertensive agents, referred to as ‘controlled RH’. The rationale for the inclusion of this category is to identify people at higher risk of adverse CVD outcomes, secondary hypertension, and antihypertensive agent-related adverse effects.

In this position statement the BIHS defines RH as uncontrolled clinic BP (≥140/90 mmHg) in people with confirmed elevated out-of-office values (average daytime ABPM or HBPM ≥ 135/85 mmHg) despite appropriate lifestyle measures and treatment with optimal (or maximum tolerated) dose of at least 3 antihypertensive drugs ideally including an ACE-I/ARB, a CCB and a thiazide-like diuretic, and exclusion of both non-adherence to antihypertensive treatment and secondary causes of hypertension (Table 1).

**Table 1:** Definition, characteristics and prevalence of RH.

BIHS RH Definition
• Uncontrolled clinic BP (≥140/90 mmHg) in people with confirmed elevated out-of-office values (average daytime ABPM or HBPM ≥ 135/85 mmHg) **AND** • Appropriate lifestyle measures **AND** • Use of optimal (or maximum tolerated dose) of at least 3 antihypertensive drugs ideally including an ACE-I/ARB, a CCB and a thiazide-like diuretic **AND** • Exclusion of both non-adherence to antihypertensive treatment and secondary causes of hypertension
**Characteristics**
RH is more common in men, older people, black-African ethnicity, low income, obstructive sleep apnoea, depression, high CVD risk, advanced hypertension-mediated organ damage, high BP at diagnosis, chronic uncontrolled BP, obesity, low physical activity, excessive alcohol and high dietary salt intake.
**Prevalence of RH**
≈ 5–10% of the hypertensive population

*RH* resistant hypertension, *BP* blood pressure, *ABPM* ambulatory blood pressure monitoring, *HBPM* home blood pressure monitoring, *ACE-I* angiotensin converting enzyme inhibitor, *ARB* angiotensin receptor blocker, *CCB* calcium channel blocker, *CVD* cardiovascular disease.

The BIHS note that people in whom BP is controlled on more than 3 antihypertensive drugs may also benefit from a review in a specialist setting. This will ensure that pseudo-resistant and secondary hypertension are excluded, and optimal drugs and doses are prescribed, thus avoiding potential over-treatment.

## Prevalence, heritability and pathophysiological aspects of RH

### Prevalence

The reported prevalence of RH varies considerably due to different definitions, clinical settings and populations studied. Moreover, the diagnosis of RH is often made incorrectly [16]. Among treated adults with hypertension the prevalence of RH is reported as between 5% and 30% [17–19]. Population-based studies often report a prevalence between 12% and 15% with a higher percentage reported when individuals at elevated CVD risk are included [20–23]. However, using the BIHS definition of RH the estimated prevalence is more likely to be nearer 5–10%.

### Heritability

Conclusive evidence regarding the heritability of RH is lacking which is unsurprising given that monogenic forms of hypertension are rare and most studies exploring candidate genes for RH have multiple methodological limitations [24–26].

### Pathophysiology

The pathophysiology of RH is complex and both inherited and environmental factors are likely to contribute to salt and water retention, which in turn contribute to volume expansion, raised peripheral resistance and increased arterial stiffness, via the interplay of neurohormonal factors including raised aldosterone, vasopressin and sympathetic activity [27]. These factors are likely to contribute to higher rates of hypertension mediated organ damage (HMOD), chronic kidney disease (CKD) and premature CVD events.

## Management of people with RH

**Fig. 1:**
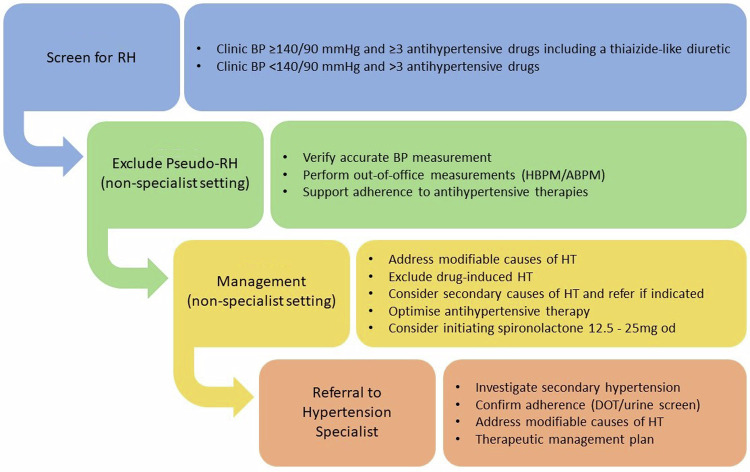
Practical approach to the investigation and management of resistant hypertension. *RH* resistant hypertension, *BP* blood pressure, *HBPM* home blood pressure monitoring, *ABPM* ambulatory blood pressure monitoring, *DOT* directly observed therapy.

The BIHS recommend a practical approach to the investigation and management of people with RH (Fig. 1).

### Screen for RH

In primary care and community-based settings, people with either suspected RH (e.g., elevated clinic BP and treatment with at least 3 antihypertensive drugs including a thiazide-like diuretic) or those at BP target on more than 3 antihypertensive drugs, can be identified by performing routine searches of electronic patient records, regular health checks, and by opportunistic encounters.

### Exclude pseudo-resistant HT

Pseudo-resistant hypertension commonly results from inaccurate BP measurement. Causes include the use of an inaccurate device or wrong sized cuff, the white-coat phenomenon, or marked brachial artery calcification (Osler phenomenon) which renders the brachial artery incompressible. Non-adherence to antihypertensive therapy is a frequent cause of pseudo-resistant hypertension. Clinicians have an important role discussing medication adherence and supporting people to identify and overcome specific barriers (e.g., complex treatment regimens and adverse drug effects) [28]. Dosette boxes are a practical tool for managing multiple medications and single-pill combinations (SPC) have been shown to improve adherence [29, 30]. Objective methods to assess medication adherence include directly observed therapy (DOT) and antihypertensive drug urine screening [31], although these are more commonly available in specialist settings. Other causes of pseudo-resistant hypertension include concurrent medications including over-the-counter remedies and substance of abuse (Fig. 1). Addressing these issues can avoid an incorrect diagnosis of RH.

#### Verify accurate blood pressure measurement

**Fig. 2:**
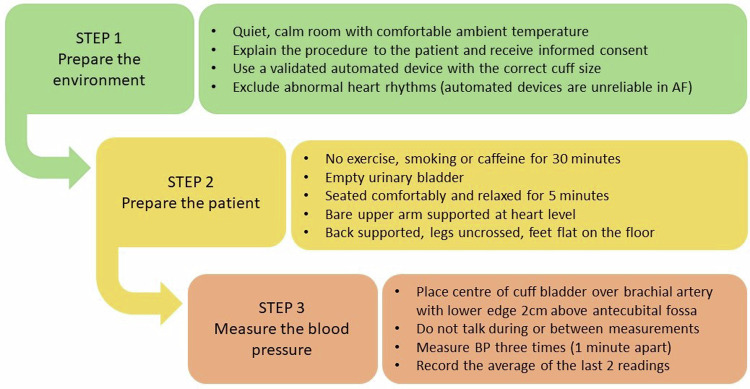
Procedure for accurate office blood pressure measurement. *AF* atrial fibrillation, *BP* blood pressure.

In people with suspected RH it is important to ensure that BP is measured correctly and elevated clinic readings are confirmed with out-of-office measurements (HBPM or ABPM) to exclude a white coat effect (Fig. 2).

##### Use of Validated Equipment

It is essential that clinically validated BP devices are used, noting that monitors over 4 years old may be inaccurate [32]. Please refer to the British and Irish Hypertension Society’s website, www.bihs.org.uk, for the current list of validated blood pressure monitoring devices.

##### Cuff size

It is important to use a cuff that is specifically made for the BP monitor and is appropriately sized for the individual [33]. The bladder length should be 75–100% of the arm circumference and the width 35–50% of the arm circumference. Poor cuff choice may lead to inaccurate diagnosis. For example, an under-sized cuff may require higher pressure to occlude blood flow in the underlying artery, leading to a false diagnosis of RH. If there is doubt over which size cuff to use, err on the side of a larger cuff as the measurement error will be significantly less than if the cuff is too small.

##### Cuff placement

The centre of the cuff bladder should be placed directly on the skin over the brachial artery at the level of the heart with the lower cuff-edge 2 cm above the antecubital fossa. Cuffs placed over clothing may overestimate BP, leading to a false diagnosis of RH.

##### Individual factors

BP can be affected by many factors including ambient noise, low temperature, stress, full bladder, recent exercise and recent (within 30 min) ingestion of caffeine or tobacco. We recommend clinic BP is measured after 3–5 min rest, with the subject seated silently with the legs unfolded, back supported and arm resting at heart height. Clothing over the upper arm should be removed and not rolled up to avoid a tourniquet effect. Talking should be avoided during and between measurements. BP should be measured at least 3 times at one-minute intervals. The average of the last 2 measurements should be recorded. In people where there is a significant difference in BP between arms, the arm with the higher measurements should be used.

##### Abnormal heart rhythms

Semi-automatic BP measurement devices are unreliable among people with abnormal heart rhythms including bradycardia, frequent extrasystoles and atrial fibrillation. In such individuals, manual BP measurement using auscultatory techniques should be used.

#### Perform out-of-office measurements (HBPM/ABPM)

We advise asking people to record their BP at home for 7 consecutive days in both the morning and evening. Appropriate training should be given and people asked to take 3 readings, one minute apart, on each occasion and the mean of the second two values recorded. If elevated, BP should be confirmed using 24 h ABPM. The BIHS (www.bihs.org.uk) and BPUK (www.bloodpressureuk.org) websites have information on BP measurement techniques.

#### Support adherence to antihypertensive therapies

Studies of non-adherence using objective measures of drugs or metabolites in blood or urine [31] have consistently shown very high rates of non-adherence (around 50%) in people with apparent RH [34–37]. Clinicians have a key role in identifying and supporting people to overcome the barriers to medication adherence. Complex drug regimens, polypharmacy, medication cost, and adverse drug effects are key contributing factor to non-adherence in RH [28]. Prescribing SPCs, where available, can improve adherence by reducing pill burden [29]. In non-specialist settings, methods to monitor adherence include self-report, questionnaires and pharmacy refill reports. In specialist settings, DOT and/or urine drug screening provide objective measures of adherence.

### Management in non-specialist settings

#### Address modifiable causes of hypertension

Several potentially modifiable factors are independently associated with RH including obesity, dietary salt intake, low physical activity, and excessive alcohol [38, 39]. It is important to note that these factors are not mutually exclusive and can be interdependent. Optimising weight, diet, and exercise are effective adjuncts to antihypertensive medications and are unanimously recommended by national and international hypertension guidelines (Table 2) [9–14]. However, adherence to lifestyle modifications remains challenging and the long-term impact on BP and CV outcomes among people with RH is currently unknown.

**Table 2:** Summary of Lifestyle Recommendations to reduce BP and CV risk (Adapted from Kulkarni et.al. *J Hum Hypertens*. 2024;38: [7] 544-54 [157] under Creative Commons licence CC BY 4.0: http://creativecommons.org/licences/by/4.0/.

**Lifestyle Recommendations to Reduce BP and CVD Risk**
1. Reduce salt to <6 g day. Pre-prepared foods like bread, breakfast cereals and ready meals may contain high salt. Check labels when shopping, swap domestic salt to a potassium enriched product and avoid adding extra salt to food. 2. Maintain a healthy weight. For people that are overweight, weight loss is associated with a reduction in blood pressure. 3. Incorporate exercise. 30 min of moderate aerobic exercise 5 times a week and resistance training 2 to 3 times a week. 4. Eat a balanced diet rich in potassium and nitrates. Include at least 5 portions (400 g) of fruit and vegetables daily. 5. Alcohol in moderation. Current guidelines recommend up to 14 units a week for men and women. Avoid binge drinking and encourage alcohol free days. 6. Avoid excess caffeine. Energy drinks with high concentrations of ingredients such as taurine and caffeine are associated with increased blood pressure [158]. 7. Stop smoking to reduce total cardiovascular risk.

*BP* blood pressure, *CVD* cardiovascular disease.

##### Obesity

Excess body fat and visceral adiposity are well established contributing factors in the development of hypertension and can also enhance salt sensitivity and promote vascular dysfunction and activation of the autonomic nervous system [40, 41]. There is a strong linear correlation between increasing Body Mass Index (BMI) and rise in BP [42–44]. Obesity is independently associated with RH [45] and the prevalence in this population is over 50% [21, 46]. In the National Health and Nutrition Examination Survey a BMI > 30 kg/m^2^ approximately doubled the risk of RH among hypertensive individuals [21]. Obesity also clusters with other risk factors and clinical features of RH including obstructive sleep apnoea (OSA)—another independent cause of raised BP [47]. Weight loss strategies most commonly combine caloric, fat or carbohydrate restriction with increased exercise. A 6–8% reduction in body weight is associated with a 5/4 mmHg reduction in SBP/ DBP, but unfortunately is difficult to maintain long-term [48–52]. The latter is unsurprising given the biological basis for obesity [53]. Indeed, obesity management has been revolutionised by GLP-1 agonists which stimulate insulin secretion, down-regulate appetite via central mechanisms and delay gastric emptying. These drugs were initially developed to manage diabetes, but their effectiveness was accompanied by significant weight loss and reduction in major adverse cardiovascular events leading to subsequent studies in non-diabetic patients [54, 55]. Weight reduction obtained by the combination of a GLP-1 agonist and glucose-dependent insulinotropic polypeptide (GIP) agonists has been compared to that produced by bariatric surgery [56]. These drugs are currently licensed in the UK for the treatment of obesity, but global supply shortages are currently limiting their use in clinical practice [57].

##### Dietary salt

Studies have clearly linked dietary salt with BP level although there is large inter-individual variation [58]. Several studies have suggested a high prevalence of excessive salt intake among people with RH [59–61]. In addition, although “salt sensitivity” is a continuous phenotype [62], the magnitude of BP rise in people with RH that can be causally attributed to dietary salt intake is considerably larger than that observed in the general hypertensive population [63]. Reducing dietary salt intake to less than 5–6 g/day per day should be encouraged in all people with hypertension. A recent meta-analysis among people on antihypertensive therapy showed a reduction in salt of 3 grams/day resulted in a decrease in BP of ≈3.5/2 mmHg [64]. This study also highlighted that salt restriction was particularly effective at reducing BP among those treated with renin angiotensin aldosterone system (RAAS) inhibitors. Evidence from randomised controlled trials (RCTs) show the beneficial effects of a low salt diet compared to a high salt diet in a small number of people with RH [63] as well as in people with CKD with prevalent RH [65]. It is important that medication reviews are conducted to ensure people are not on concurrent medication with a high salt content, to avoid iatrogenic hypertension [66, 67].

##### Dietary potassium

The landmark SSaSS trial (Salt Substitute and Stroke Study) demonstrated salt substitutes with reduced sodium and increased potassium were associated with a reduction in both BP and major adverse cardiovascular events [68]. A recent meta-analysis provides support for a population goal of potassium intake of 90 mmol per day [69]. In most trials, potassium supplementation was achieved by administration of potassium chloride pills, but the reduction in both BP and major adverse cardiovascular events was similar when dietary modifications were used [68–70]. Because potassium-rich diets tend to be heart-healthy, they are preferred over the use of pills for potassium supplementation. However, the use of potassium-enriched salt substitutes should be encouraged for home cooking.

##### Diet

The most notable among the individual strategies are the “*Dietary Approaches to Stop Hypertension*” (DASH) diet, which emphasises lean proteins, whole grains, vegetables and fruits, and low-fat dairy products [71, 72]. The DASH diet reduces BP in people with hypertension (SBP reduction of 11.6 mmHg) but also in normotension (SBP reduction of 5.3 mmHg) [71, 73]. There are however no RCTs with the DASH diet as an intervention alone specifically in RH.

##### Physical inactivity

Both reduced physical activity and lower physical fitness are independent risk factors for hypertension [74]. Although the data to show a correlation between physical inactivity and RH are limited, the prevalence of self-reported physical inactivity is high in people with RH (~40%) [45]. There is good evidence supporting the benefits of exercise therapy for hypertension [75], and several studies have reported benefits of exercise therapy among people with RH [76–83].

##### Alcohol

A causal association between alcohol consumption and increased BP has been established by RCTs and Mendelian randomisation studies [84, 85]. Around a third of people with RH have a history of excessive alcohol intake [86], and people who consume excess alcohol are more likely to develop RH during treatment for hypertension [38].

#### Exclude drug-induced hypertension

Many therapeutic agents increase BP and render hypertensive patients apparently resistant to BP lowering treatments. It is therefore essential to obtain a detailed history of all concurrent prescribed medications (considering preparations with a high salt content [66, 67]), over-the-counter products and illicit drugs. Removal or dose reduction of any such agents should be attempted where possible, although BP may not always normalise as a result. In such scenarios, multidisciplinary team working may permit alternative treatments or use of the lowest necessary doses of the interfering agent. In many cases however, medications cannot be discontinued, and appropriate antihypertensive treatment should be instated. The mechanism of action by which some therapeutic agents and other commonly used substances affect BP control are summarised in Table 3.

**Table 3:** Therapeutic agents and other substances that may affect blood pressure.

Mechanism of Action			
Sympathetic activation	Volume retention	Direct Vasoconstriction	Other
• Cocaine • Stimulants often used for ADHD, narcolepsy and hypersomnias • Decongestants • Bronchodilators • Antidepressants	• NSAIDs • Sex hormones e.g., combined contraceptive pill • Corticosteroids • Liquorice • Sodium containing antacids	• VEGF inhibitors • Calcineurin inhibitors	• Erythrocyte stimulating agents • Tyrosine kinase inhibitors • Diet supplements (Ephedra, Yohimbine, bitter orange)

*ADHD* attention deficit hyperactivity disorder, *NSAIDS* non steroidal anti-inflammatory drugs, *VEGF* vascular endothelial growth factor.

**Sympathomimetic agents** are commonly used as decongestants (e.g., phenylephrine, pseudoephedrine), as bronchodilators (e.g., salbutamol), as stimulants in attention deficit disorders (ADHD) (e.g., atomoxetine, methylphenidate, lisdexamphetamine), narcolepsy and hypersomnias (e.g., modafinil, pitosilant, sodium oxybate), as agents which increase catecholamine availability such as mono-amine oxidase inhibitors (e.g., phenelzine) and noradrenaline reuptake inhibitors (e.g., venlafaxine) and as substances of abuse (e.g., cocaine).

A recent meta-analysis of RCTs of ADHD medications revealed an average SBP rise of 2 mmHg with these agents [87]. Duration of therapy seems to be crucial with regards to hypertension. Longer (>3 years) term use of drugs such as atomoxetine, methylphenidate and lisdexamphetamine have been associated with a 4% increased risk in incident CVD for each year of treatment and a 72% increased incidence of hypertension compared to controls [88]. No increased risk was seen with shorter (<3 years) term treatment.

**Non-Steroidal Anti-Inflammatory Drugs (NSAIDs) and Steroids** may raise BP via RAAS mediated changes in renal blood flow and glomerular filtration rate resulting in sodium retention [89] particularly in salt-sensitive populations. Other mechanisms include endothelin-1 induced vasoconstriction [90] and inhibition of prostaglandin E2 and I2 [91]. NSAIDs also lower the efficacy of antihypertensive drugs and should therefore be withdrawn in people with RH if they are no longer needed, or a minimum dose used [92]. Some NSAIDs, such as sulindac may have less hypertensive effects than others [93]. The cyclo-oxygenase (COX) inhibitor celecoxib may also increase BP in a dose dependent manner [94], though less than rofecoxib especially if concomitant treatment with ACE-I or beta-blockers [95, 96].

**Corticosteroids** whether given orally, systemically or topically, may increase BP. Glucocorticoids have variable degrees of mineralocorticoid activity causing hypertension through sodium retention, direct vasoconstriction and indirect metabolic effects including weight gain and glucose intolerance. Prednisolone below a dose of 7.5 mg daily, is less likely to increase BP [97]. Fludrocortisone (a mineralocorticoid) is specifically used for raising BP in hypertensives with postural hypotension. In people with RH, reduced dosage or alternatives should be considered where possible.

**Hormone Therapy** The association between combined oral contraceptives (CoC) and hypertension is well-recognised, with studies showing a dose-dependent relationship with oestrogen strength [98]. Proposed mechanisms include increased oestrogen-mediated corticosteroid binding globulin resulting in raised cortisol [99], and upregulation of the RAAS system [100]. This risk is not seen with Progesterone-only Pills (PoP) [101]. Contraceptive options for hypertensive women include non-hormonal options, PoP and Intra-Uterine Devices (IUD). Newer formulations containing natural oestrogens and progestins with anti-mineralocorticoid effects may mitigate this link with high BP [99]. Transdermal oestrogens, commonly prescribed for menopausal symptoms may be less likely to induce hypertension than oral oestrogens [102]. Low levels of androgens in men and increased levels of androgens in women, as occurs with polycystic ovary syndrome (PCOS), are both associated with increased risk for cardiovascular disease and elevated BP [103]. The link between gender-affirming hormone therapy (GAHT) and hypertension requires further investigation [104, 105]. Anabolic steroids are often used illicitly to increase muscle bulk and power and are associated with an 8 mmHg increase in BP, a threefold increase in the prevalence of hypertension, and increased aortic stiffness [106].

**Erythropoietin** Twenty to thirty per cent of people given recombinant human erythropoietin to correct anaemia in malignancies and end-stage renal disease may have hypertension induced or exacerbated within 2 to 16 weeks of starting treatment. While polycythaemia is a recognised risk factor for hypertension (e.g., Gaisböck syndrome), erythropoietin has been shown to cause hypertension independently from elevated hematocrit. Among the proposed mechanisms are nitric oxide resistance and an increase in cytoplasmatic ionised calcium. In these individuals, calcium channel blockers are the pharmacological agent of choice [107, 108].

**Growth Factor Inhibitors** This class of agents includes Vascular Endothelial Growth Factor (VEGF) inhibitors that inhibit tumour angiogenesis such as bevacizumab and pazopanib, the latter of which also inhibits platelet derived growth factor (PDGF). Inhibition of VEGF prevents the phosphorylation of endothelial nitric oxide synthase, a potent vasodilator [109]. A recent meta-analysis showed that use of these agents was associated with an increased risk of hypertension [110]. In other studies, the risk was demonstrated to be dose dependent [111]. Indeed, the strong relationship between hypertension and VEGF inhibition may be useful as a biomarker of drug effect. Within this class are the Tyrosine Kinase Inhibitors (TKIs) that are involved in the VEGF signalling pathway. Many of these anti-cancer agents are used as targeted therapy in solid organ cancers [112]. Examples of TKIs that result in arterial hypertension due to epidermal growth factor (EGF) inhibition are gefitinib and lenvatinib.

#### Consider secondary causes of hypertension and refer if indicated

Secondary hypertension is common in people with apparent RH (≈1/3 subjects are affected depending on the definition and clinical setting) with renal diseases and primary hyperaldosteronism being the most common aetiologies [113]. Complete diagnostic workup for secondary hypertension requires an in-depth assessment which is performed in a specialist setting based on clinical characteristics of the patients and local expertise.

The BIHS recommend all people with suspected RH have a basic diagnostic workup for secondary hypertension in a non-specialist setting. A simplified screening protocol is described in Table 4. People who have clinical signs and symptoms of secondary hypertension or a positive screening test(s) should be referred to a hypertension specialist for additional tests and investigations (Table 5 and Supplementary File). This pragmatic approach to personalised medicine tailors’ investigations to the individuals’ specific characteristics and promotes the efficient use of resources.

**Table 4:** Investigations to exclude secondary hypertension.

BIHS Simplified Screening Protocol for all People with RH (non-specialist setting)
History, symptoms, physical examination and factors affecting adherence (see Table 5 and supplementary file)
Blood test including kidney function, liver enzymes, full blood count, calcium and TSH
Urine dipstick
ECG
Renal imaging (e.g., US) to detect renal scarring and other gross abnormalities
Exclude OSA
**Further Investigations Where There is Clinical Suspicion of Secondary Hypertension (usually performed in Specialist Settings)**
Renin/aldosterone and plasma/urinary metadrenalines
Urine sample for albumin:creatinine ratio and protein:creatinine ratio
24-h urinary collection for sodium and potassium
24-h urine cortisol/dexamethasone suppression test
Adrenal imaging (MRI/CT)
Assessment of renal vascularity (MRA, CTA)

*OSA* obstructive sleep apnoea, *TSH* thyroid stimulating hormone, *US* Ultrasound, *MRI* magnetic resonance imaging, *CT* Computed Tomography, *MRA* Magnetic Resonance Angiography, *CTA* Computed Tomography Angiography.

**Table 5:** Common causes of secondary hypertension, symptoms that raise clinical suspicion and recommended investigations.

Condition	Clinical Suspicion	Investigations
Kidney disease	Usually asymptomatic with only incidental finding of elevated serum creatinine or abnormalities in the urinary sediment. People with diffuse atherosclerotic disease can show a drop in eGFR after treatment with ACE-I/ARB. History of recurrent UTIs and family history of kidney disease should be evaluated.	Serum creatinine, urine dipstick, urine sample for albumin:creatinine ratio and protein:creatinine ratio. Consider renal imaging (US, MRA, CT) based on laboratory findings and clinical situation.
Primary hyperaldosteronism	Spontaneous/thiazide induced hypokalaemia, adrenal incidentaloma. Majority of cases are detected in asymptomatic individuals with normokalaemia.	Renin/aldosterone ± adrenal imaging (adrenal MRI/HRCT) followed by confirmatory tests and/or adrenal vein sampling.
Obstructive Sleep Apnoea	Snoring, daytime sleepiness, elevated BMI or neck circumference and non-dipping profile on 24-h ABPM.	Validated questionnaires such as Epworth Sleepiness Scale or STOP-BANG score. Overnight oxygen saturation monitor.
Pheochromocytoma and Paraganglioma	Paroxysmal or sustained hypertension, headaches, palpitations, hyperhidrosis, and non-CV symptoms such as weight loss or hypoglycaemia.	Plasma free or urinary fractionated metanephrines ± adrenal MRI/HRCT ± functional imaging and/or clonidine suppression test.
Cushing syndrome	Truncal obesity, striae, glucose intolerance and repeated infections.	Overnight dexamethasone suppression test and 24-h urine free cortisol. ACTH is needed to confirm hypercortisolism and imaging is required to differentiate between adrenal and pituitary causes.
Thyroid and parathyroid disorders	Usually asymptomatic: symptoms may include palpitations, tiredness, polyuria, muscle weakness, anxiety, tremor, irritability.	Evaluation of calcium, PTH, TSH and thyroid hormones. Thyroid and parathyroid ultrasound.
Aortic coarctation	Usually asymptomatic: signs may include radio-femoral delay and lower blood pressure in the lower extremities.	Aorta MRA/CT.
Further information on the secondary causes of hypertension can be found in the accompanying Supplementary File.		

*eGFR* estimated glomerular filtration rate, *ACE-I* angiotensin converting enzyme inhibitor, *ARB* angiotensin receptor blocker, *UTI* urinary tract infection, *US* ultrasound, *MRA* magnetic resonance angiography, *CT* computed tomography, *MRI* magnetic resonance imaging, *HRCT* high-resolution computed tomography, *BMI* body mass index, *ABPM* ambulatory blood pressure monitoring, *CV* cardiovascular, *ACTH* adrenocorticotropic Hormone, *TSH* thyroid stimulating hormone, *PTH* parathyroid hormone.

#### Optimise antihypertensive therapy

##### Steps 1–4: Triple Therapy

**Fig. 3:**
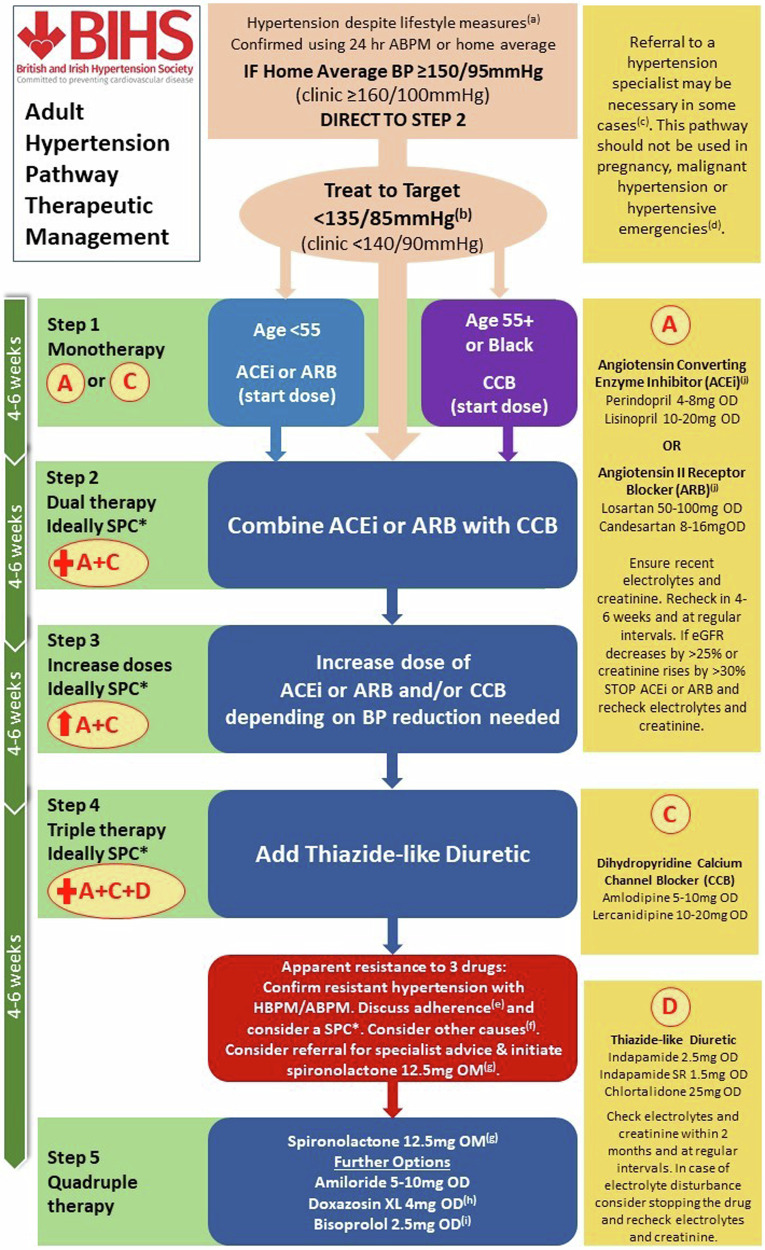
British and Irish Hypertension Society Adult Hypertension Pathway Therapeutic Management. Reproduced from Lewis et.al. *J Hum Hypertens*. 2024;38 [1]: 3-7 [114] under Creative Commons licence CC BY 4.0: http://creativecommons.org/licences/by/4.0/. Footnotes to Fig. 3: See Lewis et al. *J Hum Hypertens*. 2024;38 [1]: 3-7 for footnote explanations [114].

Steps 1–4 of the pharmacological management of RH is to ensure that the A + C + D regimen is optimised, both in terms of choice of agents and dose (Fig. 3). A practical guide to therapeutic options is available in the BIHS statement “*Adult hypertension referral pathway and therapeutic management: British and Irish Hypertension Society position statement*” [114].

**Fig. 4:**
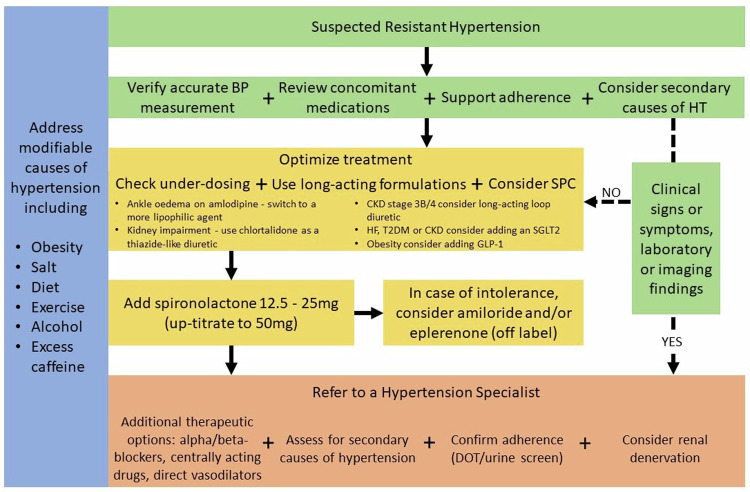
Therapeutic management of resistant hypertension. BP blood pressure, HT hypertension, CKD chronic kidney disease, HF heart failure, T2DM type 2 diabetes mellitus, DOT directly observed therapy.

In general, long-acting drugs are to be preferred, as they provide smoother BP control and are less affected by missed doses, as are agents with robust efficacy data. Under-dosing is particularly common with ACE-I/ARB, as very low doses are available for initial treatment in people with heart failure. Switching from thiazide to thiazide-like diuretic and from indapamide to chlorthalidone may be useful in people with RH and renal impairment, if eGFR > 15 mL/min/1.73 m^2^, as chlortalidone is the only thiazide-like diuretic to demonstrate efficacy in people with advanced CKD [115]. In subjects with CKD, particularly in cases of signs or symptoms suggestive of fluid retention, a long-acting loop diuretic such as torsemide [116] may be considered in addition or instead of a conventional thiazide-like agent (Fig. 4).

In people unable to tolerate standard doses of amlodipine due to ankle oedema, switching to a more lipophilic agent - such as lercanidipine or lacidipine may be helpful [117]. Alternatively, non-dihydropyridines CCB such as long-acting formulations of verapamil or diltiazem (e.g., diltiazem LA 200/300 mg) may be used, assuming there are no cardiac contra-indications.

In general, SPCs are preferred as they improve adherence and lead to better BP control, compared with single-drug equivalent combinations [29]. However, there is limited availability of suitable SPCs in the UK, as combinations generally do not contain standard doses or drugs. For example, the only dual SPC available in the UK containing indapamide has only 5 mg of perindopril, and the only triple SPC contains olmesartan, amlodipine and hydrochlorothiazide. Although hydrochlorothiazide is commonly used as monotherapy in the USA and is a component in several SPCs in the UK, it is not a first-line NICE-recommended diuretic. While often considered to be less efficacious than chlorthalidone (dose-dependent results), hydrochlorothiazide was not inferior to chlorthalidone in preventing major CV outcomes in a large pragmatic trial (albeit with some design limitations) that randomised more than 13,000 people in the Department of Veterans Affairs Health System [118]. Similar findings were reported from two other large cohort studies [119, 120]. Low-dose thiazides have no placebo-controlled RCT evidence of mortality or morbidity benefits and concerns have been raised about the risk of skin cancer with long-term use of hydrochlorothiazide, and the Medicines and Healthcare products Regulatory Agency (MHRA) has issued guidance on its use in the UK [121]. People need to be warned of the cancer risk and the need to avoid sun exposure, and it should not be used in people with a history of skin cancer [121, 122].

##### Step 5: Consider initiating Spironolactone

If BP remains elevated despite optimal triple therapy, a 4th line drug should be added. There are several different drug classes available, including alpha receptor antagonists, beta-blockers and mineralocorticoid receptor antagonists (MRA). The PATHWAY-2 RCT compared these three classes head-to-head and reported spironolactone was the most effective agent in lowering BP [123]. Spironolactone was well tolerated in the ASCOT study as a third- or fourth-line agent, with a low rate of reported side-effects [124]. These data highlight the importance of salt and water retention in RH, and support the view that aldosterone excess, that does not fulfil strict criteria for classical PA, may be responsible for resistance to commonly used antihypertensive medications [125].

Spironolactone is the only MRA licensed in the UK for RH and should be used as the 4th line drug of choice. [123, 126–128] It should be initiated at 12.5–25 mg od (if eGFR > 30 mL/min/1.73 m^2^ and K^+^ < 4.5 mmol/L) and can be up-titrated to 50 mg based on clinical response. The use of spironolactone may be limited by poor tolerability in a small number of people, such as significant hyperkalaemia, gynaecomastia or erectile dysfunction in men, and menstrual irregularities and breast tenderness in women. In such situations amiloride (5–20 mg) can be used as an alternative. In a sub-study of PATHWAY-2, people with RH were given amiloride 10–20 mg, which proved as effective in lowering BP as spironolactone, and was well tolerated [129]. Amiloride may be particularly effective in individuals with low levels of renin and aldosterone, and in black people in whom a Liddle-like phenotype appears relative common (~10–20%) [130, 131]. Eplerenone may be used as an off-label alternative in people with adverse effects due to spironolactone or amiloride. Although eplerenone is more selective (thus potentially causing fewer side effects), it is also a less potent antagonist of the mineralocorticoid receptor, and shorter acting, compared with spironolactone. In general, eplerenone must be dosed twice as high as spironolactone for therapeutic equivalence [132].

If the addition of spironolactone or equivalent is not tolerated or contra-indicated, e.g., clinically significant hyperkalaemia, then consider referral to a hypertension specialist for advice on alternative agents.

##### Step 6: Add in other drugs

There is a wide choice of additional agents available, albeit with limited RCT evidence to support widespread use, including but not limited to

- α-adreneceptor antagonists e.g., Doxazosin XL 4–8 mg.
- β-adreneceptor antagonists e.g., bisoprolol or nebivolol, both 2.5–5 mg OD.
- Centrally acting drugs e.g., methyldopa, clonidine or moxonidine
- Direct vasodilators e.g., hydralazine or minoxidil.

The choice of agent should be carefully considered for each patient, taking into consideration patient choice, previous adverse experiences and the clinical situation.

Adrenoceptor antagonists are perhaps better tolerated in general than the other classes of drug, and PATHWAY-2 provided evidence of efficacy for Doxazosin XL [123]. Therefore, they are a sensible initial choice.

Use of non-modified release doxazosin is not recommended due to higher peak concentration [133], which may produce rapid reductions in BP and is less well tolerated.

β-blockers may be considered in subjects with a heart rate ≥60 bpm with indications for their use e.g., migraine. If they are contra-indicated or poorly tolerated, α-blockers are an alternative.

Centrally acting drugs are effective antihypertensives but are associated with significant side effects such as depression, dry mouth, somnolence and insomnia. Although clonidine, a centrally acting α_2_ agonist showed a similar BP-lowering efficacy to spironolactone in RH, it appears less well tolerated [134], and requires more careful and sustained dose titration. Moreover, there is a risk of rebound hypertension during periods of nonadherence, and it must be withdrawn slowly to avoid this. Transdermal formulations could be preferred but are currently not licensed in the UK. Methyldopa is now rarely used outside pregnancy, due to its poor tolerability and challenges with dose titration. Like clonidine, it should be withdrawn slowly.

Hydralazine and minoxidil are direct acting vasodilators and can cause profound hypotension. Both are licensed in the UK for severe hypertension. Chronic use leads to fluid retention and tachycardia – so-called pseudo-tolerance – which reduces their effectiveness. Hence, they are usually given with β -blockers and loop diuretics to counteract this. Hydralazine can produce a lupus-like syndrome particularly in those genetically defined as slow acetylators, and minoxidil is associated with excess hair growth and, rarely, pericardial effusion.

### Referral to a hypertension specialist

If, after confirming the diagnosis of RH, excluding pseudo resistant HT and implementing pharmacological and non-pharmacological interventions, the BP remains above target, referral to a hypertension specialist should be considered (Fig. 1). This will enable a more detailed assessment of the secondary causes of hypertension (Tables 4, 5 and supplementary file), objective measurements of drug adherence (i.e., DOT and/or antihypertensive drug urine screening [31]) and advice on pharmacological treatment and an ongoing management plan (Fig. 4).

## Device-based treatment of RH

Renal denervation (RDN) has been developed as an interventional device-based adjunctive strategy to lower BP in individuals with uncontrolled hypertension. This therapy was developed after physiological studies demonstrated stimulation of sympathetic nerve fibres increased arterial BP [135–137]. Initial open-label trials of predominantly radio-frequency (RF) RDN showed large reductions in clinic BP [138, 139] but these results were not replicated in the first RCT [140] leading to a decade long moratorium on the use of RDN in the UK [141, 142].

Important limitations to the field in general and specifically to the methodological rigour of the initial studies led to consensus on further trial design for the so-called 2nd-generation studies, with a focus on medicines stability, ambulatory BP end-points and blinded assessments [143, 144].

Second-generation studies have confirmed that while RDN has a significant BP reduction effect, it is more modest than originally postulated and is unlikely to result in normalisation of BP values without additional anti-hypertensive therapy [145–147]. RDN has been specifically evaluated in people with RH and the available evidence suggests RDN is an overall safe procedure with renal artery damage (>50% diameter stenosis and dissection) being reported in 0.45% of people within the first 6 months [148].

The RADIANCE HTN TRIO trial investigated the addition of Ultrasound (US)RDN versus a placebo procedure to a three-medication fixed-dose combination treatment (therefore in true RH) [145]. The trial confirmed a positive effect of RDN in RH with placebo-corrected difference in ambulatory BP of ~5 mmHg at 2 months (primary end point) [145].

SPYRAL ON MED investigated RF-RDN among people on 1–3 medications (therefore not solely RH), with an initial pilot, non-powered phase of 80 people which was then used as a Bayesian informative prior and folded into an extension study (n = 337 total) of the same design. The pilot phase demonstrated a 7.4 mmHg between group difference in 24-h SBP [149]. The extension phase showed markedly different results to the pilot phase, with a large fall in BP in the placebo group and failed its primary end point of change in ambulatory BP at 6 months [146].

These results are consistent with the REQUIRE trial that used US-RDN in 142 people with RH from South Korea and Japan that again showed a large reduction in BP in the placebo group, matching that seen in the active arm [150].

Based on these findings, the BIHS supports the recent NICE guidance [IPG754] on the use of RDN in people with RH when all other therapeutic avenues have been exhausted and the patient fully understands the limitations of the procedure and the unpredictability of the effect on BP lowering. However, more robust long term data are required before the widespread use of RDN in clinical practice [151].

## Research recommendations

In recent years, new drugs have been developed that may have a role in the treatment of RH including endothelin antagonists, aldosterone synthase inhibitors, novel nsMRAs, and inhibitors of angiotensinogen production by the liver with small interfering RNA (siRNA), as well as SGLT2 inhibitors, Glucagon-like peptide-1 (GLP-1) agonists, atrial natriuretic peptides and aminopeptidase A inhibitors. Among those agents:

- The endothelial antagonist aprocitentan has proven safety and efficacy data in RH [152, 153] and was approved by the US Food and Drug Administration (FDA) for use in combination with other antihypertensive agents in adults with RH.
- Among nsMRA, finerenone has been approved in the UK for treatment of CKD and diabetes, but is not expected to play a major role in the management of RH because of its modest impact on BP.
- The angiotensin receptor/neprilysin inhibitor (ARNi) sacubitril/valsartan was initially developed for hypertension (and has been approved for this indication in Japan) but is currently only licensed for heart failure in the UK. In a post hoc subgroup analysis, sacubitril/valsartan lowered BP in adults with both heart failure with preserved ejection fraction (HFpEF) and resistant hypertension [154].
- SGLT2 inhibitors have shown favourable effects on CVD events and renal haemodynamics in people with and without type 2 diabetes, and in heart failure trials where they also lower BP [155].
- GLP-1-based agonists reduce BP and could represent an adjunctive treatment for people with hypertension and obesity [156].

There are however several gaps in the current evidence-base that require further research to optimise the investigation and management of RH (Table 6). For example, despite the availability of several pharmacological classes of antihypertensive drugs, studies assessing the efficacy of specific combinations are lacking and treatment is still not based on a personalised approach. Autonomous aldosterone production is a common feature in people with RH and further work is required to elucidate pathophysiological mechanisms. In addition, it is still unclear if, aside from hypertension control, MRAs (such as spironolactone) are superior compared to other agents (such as amiloride) in preventing major adverse cardiovascular events (MACE) in subjects with RH. The role of aldosterone synthase inhibitors and nsMRAs is still not defined in RH. SGLT2 inhibitors used in diabetes, heart failure and CKD also reduce BP and weight but are not currently licenced for BP control alone. Finally, although anti-obesity drugs such as GLP-1 agonists have revolutionised the treatment of obesity, their role in management of RH has not been extensively investigated.

**Table 6:** Research Recommendations.

**Research Questions**
1. What is the most effective drug combination for people with RH? 2. What are the pathophysiological mechanisms underpinning aldosterone production in people with RH? 3. Are MRAs (such as spironolactone) superior to other agents (such as amiloride) in preventing major adverse cardiovascular events in people with RH? 4. What is the role of aldosterone synthase inhibitors and nsMRAs in the management of people with RH? 5. What is the role of SGLT2 inhibitors and GLP-1 agonists in the management of people with RH?

*RH* resistant hypertension, *MRA* mineralocorticoid receptor antagonists, *nsMRA* non-steroidal mineralocorticoid receptor antagonists, *SGLT2* Sodium-glucose co-transporter 2, *GLP-1* glucagon-like peptide 1.

## Summary

### What is known about this topic

- People living with resistant hypertension are at high risk of adverse cardiovascular and renal outcomes.
- Management, investigations and treatment for resistant hypertension varies across the globe.

### What this study adds

- This BIHS statement provides a practical framework to facilitate the delivery of high-quality and consistent care to people living with resistant hypertension.
- We highlight important future research questions to improve outcomes for people living with resistant hypertension.

## Supplementary information

SUPPLEMENTARY INFORMATION: COMMON CAUSES OF SECONDARY HYPERTENSION

## Data Availability

All data generated and analysed for the development of this statement are included in this published article.
