# Supplementary material for: Investigation and management of resistant hypertension: British and Irish Hypertension Society position statement
Source: J Hum Hypertens. 2024 Dec 9;39(1):1–14. doi: 10.1038/s41371-024-00983-6 (PMC11717708; doi:10.1038/s41371-024-00983-6)
Supplement: Supplementary file 1 — SUPPLEMENTARY INFORMATION: COMMON CAUSES OF SECONDARY HYPERTENSION [file 41371_2024_983_MOESM1_ESM.docx]

**SUPPLEMENTARY INFORMATION: COMMON CAUSES OF SECONDARY HYPERTENSION**

1. Sleep Disturbances and Sleep-Disordered Breathing
2. Primary Hyperaldosteronism
3. Kidney Disease
   1. Disorders of the Renal Parenchyma
   2. Disorders of the Distal Renal Tubules
   3. Renovascular Disease
4. Pheochromocytoma and Paraganglioma
5. Cushing Syndrome
6. Aortic Coarctation
7. Primary Hyperparathyroidism
8. Thyroid Dysfunction
9. **SLEEP DISTURBANCES AND SLEEP-DISORDERED BREATHING**

Sleep disturbances such as insomnia, sleep fragmentation, and sleep-disordered breathing (i.e. OSA) are common contributing factors to RH and disruption of the circadian BP rhythm. (1) From a pathophysiological point of view, OSA may contribute to hypertension by increasing sympathetic nerve activation, oxidative stress, activation of the RAAS and endothelial dysfunction and haemodynamic factors associated with changes in intrathoracic pressure. (2) An association between OSA and primary aldosteronism (PA) has also been observed. (3) Indeed OSA is present in up to 80% of patients with RH and is often severe. (4, 5). One of the main components contributing to both OSA and RH is obesity. (6) Weight loss has been accompanied by improvement in OSA, RH and obesity suggesting this may be the cornerstone to the treatment of all three conditions. (7) However, most studies assessing the effect of weight loss on OSA were not conducted as RCTs, had inadequate adjustment for potential confounders or a limited follow-up period. Although improvement in OSA was observed, its resolution was uncommon and the reduction in apnoea hypopnoea index (AHI) with weight loss was unpredictable. (8, 9) Historically, the most effective way to achieve large and sustained weight loss was via bariatric surgery, however, in many cases this is either not available or unacceptable to patients and has considerable perioperative risk and long-term morbidity. Obesity management has been recently revolutionized by novel pharmacotherapies such as glucagon-like peptide 1 receptor (GLP-1) agonists; however, direct evidence for their use in OSA is limited. (10)

OSA treatment with continuous positive airway pressure (C-PAP) can improve BP control. A systematic review of 10 RCTs showed that CPAP use improved 24-hour systolic BP (-5.06 mmHg) 24-hour diastolic BP (-4.21 mmHg) and especially nocturnal systolic BP. (11) The subgroup of patients that showed the largest BP response to C-PAP use are those with a non-dipping profile, aged < 60 years, with uncontrolled hypertension and more frequent episodes of oxygen desaturation. (12, 13)

Other sleep disorders such as insomnia and short sleep duration have been associated not only with increased prevalence and incidence of hypertension and CVD events but also to RH. (14, 15, 16)

Due to the high prevalence of sleep disorders and OSA in RH, it is recommended that clinicians ask questions related to sleep duration and quality and to screen patients for symptoms of OSA (for example, Epworth Sleepiness Scale or STOP-BANG score (17)). Other indicators such as BMI, neck circumference, non-dipping profile on 24-hour ABPM should be taken into consideration.

1. **PRIMARY HYPERALDOSTERONISM**

Primary hyperaldosteronism (PA) comprise a heterogeneous group of familial and sporadic disorders characterized by a relatively autonomous renin-independent aldosterone production syndrome. PA can be classified into subtypes, that differ in terms of clinical management, including unilateral forms, which may be treated surgically (such as aldosterone-producing adenomas), and bilateral forms (such as idiopathic bilateral hyperaldosteronism) requiring medical management. (18) PA is a well characterized cause of secondary hypertension in adults with RH. (19) Considering a prevalence exceeding 10% in patients with RH, PA should be systematically evaluated in this population regardless of other clinical features and normal serum potassium levels. Apart from being a potentially treatable form of hypertension, PA may also be an independent CVD risk factor as those affected display an increased CVD risk (including stroke, coronary artery disease, atrial fibrillation, heart failure, diabetes and metabolic syndrome), which is not fully explained by BP level. (20) The challenge for clinicians managing RH is the complexity of screening individuals whose BP is uncontrolled on 3 antihypertensive agents affecting the RAAS and are often unsuitable for a complete washout. In the setting of RH, we therefore endorse a pragmatic approach described below:

1. Before evaluating the aldosterone/renin ratio (ARR), consider discontinuing β-adrenergic antagonists and centrally-acting drugs (such as clonidine and alpha-methyldopa), which suppress renin release and increase the ratio of false positive results. Spironolactone should also be discontinued but all other antihypertensive medications can be continued during the screen for PA since knowing the effects of different drug classes on ARR and its components can assist in the decision-making process.
2. If the ARR is elevated and/or renin is suppressed despite the interfering effects of medications which stimulate the RAAS (including ACE-I/ ARB and diuretics) perform adrenal imaging and consider selective washout for patients who may benefit from further investigations (i.e. confirmation tests and adrenal vein sampling).
3. If renin is not suppressed and ARR is not elevated, PA is unlikely and unless there is a high pre-test probability of PA (such as hypokalaemia or an adrenal adenoma incidentaloma) medical management of hypertension should be prioritized.
4. In case of adrenal abnormalities, selective washout can be considered for adrenal vein sampling.

Details related to investigations and management of PA can be found in “*Diagnosis and management of primary hyperaldosteronism in patients with hypertension: a practical approach endorsed by the British and Irish Hypertension Society*”. (18)

1. **KIDNEY DISEASE**

Chronic kidney disease (CKD) is a common cause of RH, with prevalence and severity of hypertension rising steadily with progressive renal failure. (21, 22, 23) RH among patients with CKD is related to a decline in glomerular filtration rate with reduced clearance of solutes, impaired pressure natriuresis, and extracellular volume expansion; activation of RAAS, and promotion of vasoconstrictors (such as endothelin) over vasodilators (such as nitric oxide); and small vessel disease with reduced vascular compliance, accelerated atherosclerosis, and media calcification in the context of hyperparathyroidism and deranged calcium/phosphate homeostasis. CKD is a strong independent CVD risk factor, and, like hypertension, is asymptomatic in its early stages. All patients with RH should be screened for CKD and renal causes of secondary hypertension. ABPM is preferable over home readings as CKD is associated with an increased risk of nocturnal non-dipping and masked hypertension. (24, 25). Renal causes of hypertension are commonly divided into disorders of the renal parenchyma, disorders of the distal renal tubules and renovascular disease.

**3.1 DISORDERS OF THE RENAL PARENCHYMA**

Disease of renal parenchyma remains the most common cause of hypertension (up to 5% of total cases) and of secondary hypertension (up to 50-75%). (26) Common clinical presentations include:

**Renal Scarring** is a non-specific relatively common finding among young adult with hypertension, often following a history of recurrent urinary tract infections/ pyelonephritis in childhood or vesicoureteral reflux. Reduction in renal function can be subtle and proteinuria absent. In cases of evidence of hyperreninemic hyperaldosteronism, RAAS blockers including spironolactone should be considered once renal artery stenosis has been excluded.

Congenital solitary kidneys, horseshoe kidney, unilateral hypoplastic kidney with a normal contralateral kidney are conditions that may be associated with hypertension. In some cases, nuclear renography may demonstrate that one small (hypoplastic or scarred) kidney is contributing little to total renal function. In such cases, robotic/laparascopic nephrectomy may be considered following MDT discussion

**Autosomal Dominant Polycystic Kidney Disease (ADPKD)** Hypertension is common in ADPKD, usually in the second and third decade of life and before any detectable renal function decline and typically without proteinuria. The proposed driver for hypertension is increased renin release following ischaemia induced by cyst expansion. The disease is usually diagnosed on renal ultrasound (and can be confirmed with genetic testing). Due to the pathogenetic mechanisms, ACE-I or ARBs are the antihypertensive agents of choice. Tolvaptan has recently been introduced into clinical practice for the management of ADPKD and may be associated with long term BP reduction. (27)

**Glomerular and Tubulo-interstitial Diseases** are usually present with either of the “renal syndromes” (nephrotic, nephritic, rapidly progressive glomerulonephritis or isolated urinary abnormalities), and when suspected should trigger a referral to a nephrologist as the identification and treatment of the underlying renal disease is key to both hypertension management and prevention of renal function loss.

Investigations for renal disease should include:

1. Urine tests: Urine dipstick (for haematuria, proteinuria or leukocyturia) and quantification of proteinuria on laboratory assays. Albumin to creatinine ratio (ACR) can detect clinically significant lower levels of glomerular proteinuria, arguably more relevant in detecting hypertension mediated organ damage (HMOD). Total protein to creatinine ratio (PCR) on the other hand may allow detection of tubular and overflow proteinuria, hence both should be performed in screening for renal disease. (28)
2. Renal function assessment: serum creatinine and estimated glomerular filtration rate (eGFR) can be misleading in the early stages of chronic renal disease or in cases of extreme (high or low) muscle mass. When a reduction in renal function remains strongly suspected, estimation with alternative endogenous markers (such as cystatin C) or direct measurements with radio-isotopes (renal scintigraphy) should be considered.
3. Renal imaging: Ultrasonography represents the initial screening test. Kidney MRI/renal magnetic resonance angiography (MRA) should be considered as part of an integrated work-up for hypertension (extending to the study of renal parenchyma and vasculature, adrenal glands and aorta).

**3.2 DISORDERS OF THE DISTAL RENAL TUBULES**

Monogenic disorders can affect renal tubular epithelial cell function. Mostly asymptomatic, they often present with early onset hypertension in childhood, often in the context of a family history of early onset hypertension. Despite rare, once diagnosed these syndromes (including but not limited to Gordon syndrome, Liddle syndrome, and Geller syndrome) respond well to specific treatments. Suspicion is raised by low renin and low/normal aldosterone and electrolyte abnormalities. Such patients should be referred to a hypertension specialist for genetic testing and management advice.

**3.3 RENOVASCULAR DISEASE**

Renovascular hypertension is the consequence of RAAS activation following renal artery stenosis that resolves with restoration of arterial patency. Clinical presentation, treatment options and prognosis depend on the aetiology and the most common scenarios include:

**Atheromatous Renal Artery Disease** represents almost 90% of renal artery stenosis in developed countries with a prevalence of 7% among those aged >65 years in the general population. (29) It is a relatively common cause of RH (8 – 14%) and is usually subclinical. It is more frequent among smokers, those with dyslipidaemia, coronary artery disease, peripheral vascular disease and cerebrovascular disease. (30) Other presentations include ischemic nephropathy, acute kidney injury following RAAS blockade and flushing pulmonary oedema. It can be suspected on imaging as it usually affects the proximal (ostial) renal artery and renal or epigastric bruits may be heard on examination. The presence of diffuse atheromatic disease elsewhere supports the diagnosis.

**Fibromuscular Disease (FMD)** represents nearly all the remaining 10% of cases. FMD is defined as an idiopathic, non-atherosclerotic, non-inflammatory disease of the arterial wall musculature, causing stenosis, aneurysms or dissections of small and medium arteries. (31) It affects relatively more women and more commonly involves renal and carotid arteries. Symptoms at presentation are associated with carotid or vertebral artery disease (including stroke following dissection) or renal artery disease (including renovascular hypertension and renal infarction). (32) Occasionally it presents as spontaneous coronary artery dissection (up to 4% of all acute coronary syndromes). (33, 34) Non-symptomatic cases may be significantly more common. (35) FMD frequently affects multiple sites, so that vascular screening is strongly advised, and in particular cervical studies should be offered to individuals with renal artery involvement. (36)

**Other Rare Causes** include vasculitis and mid aortic syndrome. The latter usually presents in childhood with severe hypertension and is characterised by stenosis of the abdominal aorta. This is often associated with stenosis of the renal and/or splanchnic arteries and is detectable on renovascular imaging.

The clinical suspicion of renovascular hypertension should be raised by any of the following: RH, hypertension emergencies, biochemical features of hyperaldosteronism (low potassium with metabolic alkalosis), significant change in renal function (creatinine rise > 30%) following initiation of RAAS blockade, renal asymmetry. Despite the RAAS driving renovascular hypertension and some evidence on its role in predicting response to treatment, renin and aldosterone measurements have no use in the diagnosis of renovascular hypertension. (37, 38, 39, 40)

A stenosis is considered hemodynamically significant if it causes at least a 60% reduction in the luminal diameter (equivalent to an 80% reduction in luminal area). (41) Digital Subtraction Angiography is the gold standard, but CT angiogram and MR are excellent non-invasive first line tests, both with high diagnostic performance and most importantly negative predictive value (sensitivity, specificity, and positive and negative predictive values respectively of 94%, 93%, 71%, and 99% for CTA and 90%, 94%, 75%, and 98% for gadolinium-enhanced MRA). (42) MR, despite the lower sensitivity and positive predictive value, may be the screening modality of choice in young patients and in the elderly with renal failure to avoid the exposure to radiation and iodinated contrast. Doppler ultrasound (US), despite more widely available, requires specific expertise and is operator dependent.

Treatment options include percutaneous and surgical revascularisation and medical treatment. Following a number of landmark trials that failed to demonstrate a benefit from percutaneous revascularisation in atherosclerotic RAS (29, 43) , most guidelines (44, 45, 46, 47, 48, 49, 50, 51, 52, 53) recommend considering revascularization only in specific situations and referral to a specialist hypertension service should be considered.

**4.0 PHEOCHROMOCYTOMA AND PARAGANGLIOMA**

Pheochromocytoma and Paraganglioma are rare tumours arising respectively from adrenal and extra-adrenal chromaffin cells. Their prevalence in the general hypertensive population has been reported to be between 0.2 and 0.6%. (54, 55, 56) Among patients with RH, their prevalence is probably higher (0.7 to 4%). (57) These conditions are associated with higher CV morbidity and mortality. They can metastasize, and in rare cases large masses can lead to the compression of other organs. Moreover, some patients carry germline genetic mutations requiring cascade screening of family members.

Suspicious symptoms include paroxysmal or sustained hypertension, headaches, palpitations, hyperhidrosis, and non-CV symptoms such as weight loss or hypoglycaemia. In some patients with pheochromocytoma, exposure to medications including opiates, metoclopramide, beta blockers (in the absence of alpha-blockade), noradrenaline reuptake inhibitors (e.g. venlafaxine, tricyclic antidepressants, cocaine) and corticosteroids can precipitate a hypertensive crisis. However, pheochromocytomas may also be asymptomatic and present as incidentalomas. (58)

Biochemical screening can be performed by measuring plasma free or urinary fractionated metanephrines. Both tests have high sensitivity and specificity. (59) As the pre-test probability of pheochromocytoma is very low, there is a relatively high probability of false positive results, likely due to interfering medications or conditions such as OSA or obesity. Adrenal and functional imaging (such as Magnetic Resonance Imaging (MRI) / Computed Tomography (CT) and Nuclear scans with agents such as ^68^Ga-DOTATATE , ^18^F-FDG, ^18^F-FDOPA and 131I-MIBG) can be used to identify primary and metastatic tumours and should be considered where a clonidine suppression test gives inconclusive results. (60)

Treatment is surgical with surgical preparation including treatment with α-adrenergic blockade, high-sodium and fluid intake to correct volume depletion to prevent intraoperative BP instability. Metastatic disease may be effectively treated with targeted radionuclide therapy using agents including ^131^I-MIBG and ^177^Lu-DOTATATE. (61)

Long term follow-up with regular measurements of metanephrines is recommended due to the risk of metastases and recurrence. In a relatively large cohort study, 74% of patients were normotensive at 5 years and 45% at 10 years. (62)

**5.0 CUSHING SYNDROME**

Cushing syndrome is characterised by chronic exogenous or endogenous corticosteroid excess. Its clinical presentation includes features of severe metabolic syndrome with hypertension, visceral obesity, impairment of glucose tolerance, dyslipidaemia, but also hirsutism, menstrual disturbances, proximal muscle weakness, changes in body habitus and psychiatric symptoms. Endogenous Cushing syndrome is extremely rare with an incidence of 0.7–2.4 per million population per year. (63) Its prevalence in RH is unknown. Hypertension is present in about 80% of patients, it is usually more severe in adrenal subtypes and there is no correlation with cortisol levels. Several mechanisms are involved in the pathophysiology of hypertension in Cushing syndrome, including activation of the RAAS, mineralocorticoid effect of glucocorticoid, vasoconstriction, and co-presence of sleep apnoea. (64)

Initial screening includes overnight dexamethasone suppression test, late night salivary cortisol or 24-hour urine free cortisol measurements in duplicate. Measurement of Adrenocorticotropic Hormone (ACTH) is needed to confirm hypercortisolism and imaging is required to differentiate between adrenal and pituitary causes.

Surgical or medical treatment of responsible lesions is associated with reduced BP and CV risk; however, higher CV risk may persist and up to 25% of patients may have residual hypertension requiring ongoing medical treatment. (65) In cases of medical management of Cushing syndrome, early use of mineralocorticoid receptor antagonists should be considered to control BP.

**6.0 AORTIC COARCTATION**

Aortic coarctation may cause RH if it remains undetected. The presentation and manifestation will depend on the severity of narrowing and time of diagnosis. Most patients are diagnosis early in life and presentation in adults is relatively rare, with only ≈10% of patients with aortic coarctation being diagnosed after age 40 years. (66) Suspicion may be raised if there is a family history of aortopathy and bicuspid aortic valve disease. Clinical indications that warrant further investigation include upper extremity hypertension with lower BP in the lower extremities and delayed femoral pulses. Investigations include imaging of the aorta. Treatment may be required by balloon angioplasty or reconstructive surgery.

**7.0 PRIMARY HYPERPARATHYROIDISM**

Around 60 to 75% of patients with primary hyperparathyroidism (PHPT) are hypertensive. (67, 68) Conversely, the prevalence of PHPT in the general hypertensive population is very low (<0.5%). (69) The exact prevalence of PHPT in the RH population is unknown, but it is likely to be higher. PHPT is associated with increased CV mortality. (70)

We recommend screening all patients with RH with a bone profile and, if hypercalcaemic, to further investigate for PHPT (**Table 2**). Of note, while interfering medications causing hypercalcaemia such as thiazide and thiazide-like diuretics should be considered (71) about a quarter of patients with thiazide associated hypercalcaemia have PHPT. (72)

**8.0 THYROID DYSFUNCTION:**

Hypothyroidism (73) and hyperthyroidism (74) are known causes of secondary hypertension, and their presence increases overall CVD risk (75). Less than one percent of secondary hypertension is caused by thyroid dysfunction. (53) The prevalence of thyroid dysfunction in the RH population has not been reported. In addition, both subclinical hyperthyroidism (76) and subclinical hypothyroidism (77) are associated with adverse CV outcomes. This is likely to increase overall CV risk in patients with RH. We recommend screening all patients with RH for thyroid dysfunction with a TSH level.

**REFERENCES**

1. Huart J, Persu A, Lengele JP, Krzesinski JM, Jouret F, Stergiou GS. Pathophysiology of the Nondipping Blood Pressure Pattern. Hypertension. 2023;80(4):719-29.

2. Phillips CL, O'Driscoll DM. Hypertension and obstructive sleep apnea. Nat Sci Sleep. 2013;5:43-52.

3. Zhang R, Cai X, Lin C, Yang W, Lv F, Han X, et al. Primary aldosteronism and obstructive sleep apnea: A meta-analysis of prevalence and metabolic characteristics. Sleep Med. 2024;114:8-14.

4. Lloberes P, Lozano L, Sampol G, Romero O, Jurado MJ, Rios J, et al. Obstructive sleep apnoea and 24-h blood pressure in patients with resistant hypertension. J Sleep Res. 2010;19(4):597-602.

5. Thunstrom E, Manhem K, Rosengren A, Peker Y. Blood Pressure Response to Losartan and Continuous Positive Airway Pressure in Hypertension and Obstructive Sleep Apnea. Am J Respir Crit Care Med. 2016;193(3):310-20.

6. Jehan S, Zizi F, Pandi-Perumal SR, Wall S, Auguste E, Myers AK, et al. Obstructive Sleep Apnea and Obesity: Implications for Public Health. Sleep Med Disord. 2017;1(4).

7. Romero-Corral A, Caples SM, Lopez-Jimenez F, Somers VK. Interactions between obesity and obstructive sleep apnea: implications for treatment. Chest. 2010;137(3):711-9.

8. Edwards BA, Bristow C, O'Driscoll DM, Wong AM, Ghazi L, Davidson ZE, et al. Assessing the impact of diet, exercise and the combination of the two as a treatment for OSA: A systematic review and meta-analysis. Respirology. 2019;24(8):740-51.

9. Wong AM, Barnes HN, Joosten SA, Landry SA, Dabscheck E, Mansfield DR, et al. The effect of surgical weight loss on obstructive sleep apnoea: A systematic review and meta-analysis. Sleep Med Rev. 2018;42:85-99.

10. Blackman A, Foster GD, Zammit G, Rosenberg R, Aronne L, Wadden T, et al. Effect of liraglutide 3.0 mg in individuals with obesity and moderate or severe obstructive sleep apnea: the SCALE Sleep Apnea randomized clinical trial. Int J Obes (Lond). 2016;40(8):1310-9.

11. Labarca G, Schmidt A, Dreyse J, Jorquera J, Enos D, Torres G, et al. Efficacy of continuous positive airway pressure (CPAP) in patients with obstructive sleep apnea (OSA) and resistant hypertension (RH): Systematic review and meta-analysis. Sleep Med Rev. 2021;58:101446.

12. Pengo MF, Oscullo G, Gomez-Olivas JD, Bilo G, Parati G, Martinez-Garcia MA. Nocturnal BP Profile Predicts CPAP Effect on BP in Patients With OSA and Resistant Hypertension. Chest. 2023;164(5):1302-4.

13. Pengo MF, Soranna D, Giontella A, Perger E, Mattaliano P, Schwarz EI, et al. Obstructive sleep apnoea treatment and blood pressure: which phenotypes predict a response? A systematic review and meta-analysis. Eur Respir J. 2020;55(5).

14. Friedman O, Bradley TD, Ruttanaumpawan P, Logan AG. Independent association of drug-resistant hypertension to reduced sleep duration and efficiency. Am J Hypertens. 2010;23(2):174-9.

15. Itani O, Jike M, Watanabe N, Kaneita Y. Short sleep duration and health outcomes: a systematic review, meta-analysis, and meta-regression. Sleep Med. 2017;32:246-56.

16. Johnson KA, Gordon CJ, Chapman JL, Hoyos CM, Marshall NS, Miller CB, et al. The association of insomnia disorder characterised by objective short sleep duration with hypertension, diabetes and body mass index: A systematic review and meta-analysis. Sleep Med Rev. 2021;59:101456.

17. Chung F, Abdullah HR, Liao P. STOP-Bang Questionnaire: A Practical Approach to Screen for Obstructive Sleep Apnea. Chest. 2016;149(3):631-8.

18. Faconti L, Kulkarni S, Delles C, Kapil V, Lewis P, Glover M, et al. Diagnosis and management of primary hyperaldosteronism in patients with hypertension: a practical approach endorsed by the British and Irish Hypertension Society. J Hum Hypertens. 2024;38(1):8-18.

19. Douma S, Petidis K, Doumas M, Papaefthimiou P, Triantafyllou A, Kartali N, et al. Prevalence of primary hyperaldosteronism in resistant hypertension: a retrospective observational study. Lancet. 2008;371(9628):1921-6.

20. Milliez P, Girerd X, Plouin PF, Blacher J, Safar ME, Mourad JJ. Evidence for an increased rate of cardiovascular events in patients with primary aldosteronism. J Am Coll Cardiol. 2005;45(8):1243-8.

21. Tozawa M, Iseki K, Iseki C, Kinjo K, Ikemiya Y, Takishita S. Blood pressure predicts risk of developing end-stage renal disease in men and women. Hypertension. 2003;41(6):1341-5.

22. Cai G, Zheng Y, Sun X, Chen X, Survey of Prevalence A, Treatment Rates in Chronic Kidney Disease Patients with Hypertension in China Collaborative G. Prevalence, awareness, treatment, and control of hypertension in elderly adults with chronic kidney disease: results from the survey of Prevalence, Awareness, and Treatment Rates in Chronic Kidney Disease Patients with Hypertension in China. J Am Geriatr Soc. 2013;61(12):2160-7.

23. Klag MJ, Whelton PK, Randall BL, Neaton JD, Brancati FL, Ford CE, et al. Blood pressure and end-stage renal disease in men. N Engl J Med. 1996;334(1):13-8.

24. Drawz PE, Alper AB, Anderson AH, Brecklin CS, Charleston J, Chen J, et al. Masked Hypertension and Elevated Nighttime Blood Pressure in CKD: Prevalence and Association with Target Organ Damage. Clin J Am Soc Nephrol. 2016;11(4):642-52.

25. Drawz PE, Beddhu S, Kramer HJ, Rakotz M, Rocco MV, Whelton PK. Blood Pressure Measurement: A KDOQI Perspective. Am J Kidney Dis. 2020;75(3):426-34.

26. Preston RA, Epstein M. Renal parenchymal disease and hypertension. Semin Nephrol. 1995;15(2):138-51.

27. Heida JE, Gansevoort RT, Torres VE, Devuyst O, Perrone RD, Lee J, et al. The Effect of Tolvaptan on BP in Polycystic Kidney Disease: A Post Hoc Analysis of the TEMPO 3:4 Trial. J Am Soc Nephrol. 2021;32(7):1801-12.

28. Chang DR, Yeh HC, Ting IW, Lin CY, Huang HC, Chiang HY, et al. The ratio and difference of urine protein-to-creatinine ratio and albumin-to-creatinine ratio facilitate risk prediction of all-cause mortality. Sci Rep. 2021;11(1):7851.

29. Cooper CJ, Murphy TP, Cutlip DE, Jamerson K, Henrich W, Reid DM, et al. Stenting and medical therapy for atherosclerotic renal-artery stenosis. N Engl J Med. 2014;370(1):13-22.

30. Lao D, Parasher PS, Cho KC, Yeghiazarians Y. Atherosclerotic renal artery stenosis--diagnosis and treatment. Mayo Clin Proc. 2011;86(7):649-57.

31. Faconti L, Morselli F, Sinha M, Chrysochou C, Chowienczyk PJ, British, et al. Fibromuscular dysplasia and hypertension-a statement on behalf of the British and Irish Hypertension Society. J Hum Hypertens. 2021;35(11):1051-3.

32. Kim ESH, Olin JW, Froehlich JB, Gu X, Bacharach JM, Gray BH, et al. Clinical manifestations of fibromuscular dysplasia vary by patient sex: a report of the United States registry for fibromuscular dysplasia. J Am Coll Cardiol. 2013;62(21):2026-8.

33. Adlam D, Olson TM, Combaret N, Kovacic JC, Iismaa SE, Al-Hussaini A, et al. Association of the PHACTR1/EDN1 Genetic Locus With Spontaneous Coronary Artery Dissection. J Am Coll Cardiol. 2019;73(1):58-66.

34. Djokovic A, Krljanac G, Matic P, Zivic R, Djulejic V, Marjanovic Haljilji M, et al. Pathophysiology of spontaneous coronary artery dissection: hematoma, not thrombus. Front Cardiovasc Med. 2023;10:1260478.

35. Plouin PF, Perdu J, La Batide-Alanore A, Boutouyrie P, Gimenez-Roqueplo AP, Jeunemaitre X. Fibromuscular dysplasia. Orphanet J Rare Dis. 2007;2:28.

36. Plouin PF, Baguet JP, Thony F, Ormezzano O, Azarine A, Silhol F, et al. High Prevalence of Multiple Arterial Bed Lesions in Patients With Fibromuscular Dysplasia: The ARCADIA Registry (Assessment of Renal and Cervical Artery Dysplasia). Hypertension. 2017;70(3):652-8.

37. Covic A, Gusbeth-Tatomir P. The role of the renin-angiotensin-aldosterone system in renal artery stenosis, renovascular hypertension, and ischemic nephropathy: diagnostic implications. Prog Cardiovasc Dis. 2009;52(3):204-8.

38. Daidoji H, Tamada Y, Suzuki S, Watanabe K, Shikama T, Kikuchi Y, et al. Plasma Renin Activity Predicts the Improvement in Resistant Hypertension after Percutaneous Transluminal Renal Artery Angioplasty. Intern Med. 2016;55(23):3421-6.

39. Gomez JA. Renin Angiotensin Aldosterone System Functions in Renovascular Hypertension. In: McFarlane SI, editor. Renin-Angiotensin Aldosterone System. Rijeka: IntechOpen; 2021.

40. Kotliar C, Inserra F, Forcada P, Cavanagh E, Obregon S, Navari C, et al. Are plasma renin activity and aldosterone levels useful as a screening test to differentiate between unilateral and bilateral renal artery stenosis in hypertensive patients? J Hypertens. 2010;28(3):594-601.

41. Persu A, Giavarini A, Touze E, Januszewicz A, Sapoval M, Azizi M, et al. European consensus on the diagnosis and management of fibromuscular dysplasia. J Hypertens. 2014;32(7):1367-78.

42. Rountas C, Vlychou M, Vassiou K, Liakopoulos V, Kapsalaki E, Koukoulis G, et al. Imaging modalities for renal artery stenosis in suspected renovascular hypertension: prospective intraindividual comparison of color Doppler US, CT angiography, GD-enhanced MR angiography, and digital substraction angiography. Ren Fail. 2007;29(3):295-302.

43. Astral_Investigators, Wheatley K, Ives N, Gray R, Kalra PA, Moss JG, et al. Revascularization versus medical therapy for renal-artery stenosis. N Engl J Med. 2009;361(20):1953-62.

44. Aboyans V, Ricco JB, Bartelink MEL, Bjorck M, Brodmann M, Cohnert T, et al. 2017 ESC Guidelines on the Diagnosis and Treatment of Peripheral Arterial Diseases, in collaboration with the European Society for Vascular Surgery (ESVS): Document covering atherosclerotic disease of extracranial carotid and vertebral, mesenteric, renal, upper and lower extremity arteriesEndorsed by: the European Stroke Organization (ESO)The Task Force for the Diagnosis and Treatment of Peripheral Arterial Diseases of the European Society of Cardiology (ESC) and of the European Society for Vascular Surgery (ESVS). Eur Heart J. 2018;39(9):763-816.

45. Kane GC, Xu N, Mistrik E, Roubicek T, Stanson AW, Garovic VD. Renal artery revascularization improves heart failure control in patients with atherosclerotic renal artery stenosis. Nephrol Dial Transplant. 2010;25(3):813-20.

46. Klein AJ, Jaff MR, Gray BH, Aronow HD, Bersin RM, Diaz-Sandoval LJ, et al. SCAI appropriate use criteria for peripheral arterial interventions: An update. Catheter Cardiovasc Interv. 2017;90(4):E90-E110.

47. Messerli FH, Bangalore S, Makani H, Rimoldi SF, Allemann Y, White CJ, et al. Flash pulmonary oedema and bilateral renal artery stenosis: the Pickering syndrome. Eur Heart J. 2011;32(18):2231-5.

48. Pickering TG, Herman L, Devereux RB, Sotelo JE, James GD, Sos TA, et al. Recurrent pulmonary oedema in hypertension due to bilateral renal artery stenosis: treatment by angioplasty or surgical revascularisation. Lancet. 1988;2(8610):551-2.

49. Ritchie J, Green D, Chrysochou C, Chalmers N, Foley RN, Kalra PA. High-risk clinical presentations in atherosclerotic renovascular disease: prognosis and response to renal artery revascularization. Am J Kidney Dis. 2014;63(2):186-97.

50. Umemura S, Arima H, Arima S, Asayama K, Dohi Y, Hirooka Y, et al. The Japanese Society of Hypertension Guidelines for the Management of Hypertension (JSH 2019). Hypertens Res. 2019;42(9):1235-481.

51. van Jaarsveld BC, Krijnen P, Pieterman H, Derkx FH, Deinum J, Postma CT, et al. The effect of balloon angioplasty on hypertension in atherosclerotic renal-artery stenosis. Dutch Renal Artery Stenosis Intervention Cooperative Study Group. N Engl J Med. 2000;342(14):1007-14.

52. Vassallo D, Ritchie J, Green D, Chrysochou C, Kalra PA. The effect of revascularization in patients with anatomically significant atherosclerotic renovascular disease presenting with high-risk clinical features. Nephrol Dial Transplant. 2018;33(3):497-506.

53. Whelton PK, Carey RM, Aronow WS, Casey DE, Jr., Collins KJ, Dennison Himmelfarb C, et al. 2017 ACC/AHA/AAPA/ABC/ACPM/AGS/APhA/ASH/ASPC/NMA/PCNA Guideline for the Prevention, Detection, Evaluation, and Management of High Blood Pressure in Adults: A Report of the American College of Cardiology/American Heart Association Task Force on Clinical Practice Guidelines. Hypertension. 2018;71(6):e13-e115.

54. Anderson GH, Jr., Blakeman N, Streeten DH. The effect of age on prevalence of secondary forms of hypertension in 4429 consecutively referred patients. J Hypertens. 1994;12(5):609-15.

55. Ariton M, Juan CS, AvRuskin TW. Pheochromocytoma: clinical observations from a Brooklyn tertiary hospital. Endocr Pract. 2000;6(3):249-52.

56. Sinclair AM, Isles CG, Brown I, Cameron H, Murray GD, Robertson JW. Secondary hypertension in a blood pressure clinic. Arch Intern Med. 1987;147(7):1289-93.

57. Martell N, Rodriguez-Cerrillo M, Grobbee DE, Lopez-Eady MD, Fernandez-Pinilla C, Avila M, et al. High prevalence of secondary hypertension and insulin resistance in patients with refractory hypertension. Blood Press. 2003;12(3):149-54.

58. Aggarwal S, Prete A, Chortis V, Asia M, Sutcliffe RP, Arlt W, et al. Pheochromocytomas Most Commonly Present As Adrenal Incidentalomas: A Large Tertiary Center Experience. J Clin Endocrinol Metab. 2023;109(1):e389-e96.

59. Schwartz GL. Screening for adrenal-endocrine hypertension: overview of accuracy and cost-effectiveness. Endocrinol Metab Clin North Am. 2011;40(2):279-94, vii.

60. Carrasquillo JA, Chen CC, Jha A, Ling A, Lin FI, Pryma DA, et al. Imaging of Pheochromocytoma and Paraganglioma. J Nucl Med. 2021;62(8):1033-42.

61. Prado-Wohlwend S, Del Olmo-Garcia MI, Bello-Arques P, Merino-Torres JF. Response to targeted radionuclide therapy with [(131)I]MIBG AND [(177)Lu]Lu-DOTA-TATE according to adrenal vs. extra-adrenal primary location in metastatic paragangliomas and pheochromocytomas: A systematic review. Front Endocrinol (Lausanne). 2022;13:957172.

62. Plouin PF, Chatellier G, Fofol I, Corvol P. Tumor recurrence and hypertension persistence after successful pheochromocytoma operation. Hypertension. 1997;29(5):1133-9.

63. Sharma ST, Nieman LK, Feelders RA. Cushing's syndrome: epidemiology and developments in disease management. Clin Epidemiol. 2015;7:281-93.

64. Isidori AM, Graziadio C, Paragliola RM, Cozzolino A, Ambrogio AG, Colao A, et al. The hypertension of Cushing's syndrome: controversies in the pathophysiology and focus on cardiovascular complications. J Hypertens. 2015;33(1):44-60.

65. Nieman LK, Biller BM, Findling JW, Murad MH, Newell-Price J, Savage MO, et al. Treatment of Cushing's Syndrome: An Endocrine Society Clinical Practice Guideline. J Clin Endocrinol Metab. 2015;100(8):2807-31.

66. Liberthson RR, Pennington DG, Jacobs ML, Daggett WM. Coarctation of the aorta: review of 234 patients and clarification of management problems. Am J Cardiol. 1979;43(4):835-40.

67. Graff-Baker AN, Bridges LT, Chen Q, Faries MB, Said M. Parathyroidectomy for Patients With Primary Hyperparathyroidism and Associations With Hypertension. JAMA Surg. 2020;155(1):32-9.

68. Kalla A, Krishnamoorthy P, Gopalakrishnan A, Garg J, Patel NC, Figueredo VM. Primary hyperparathyroidism predicts hypertension: Results from the National Inpatient Sample. Int J Cardiol. 2017;227:335-7.

69. Berglund G, Andersson O, Wilhelmsen L. Prevalence of primary and secondary hypertension: studies in a random population sample. Br Med J. 1976;2(6035):554-6.

70. Seib CD, Meng T, Cisco RM, Suh I, Lin DT, Harris AHS, et al. Adverse Cardiovascular Outcomes Among Older Adults With Primary Hyperparathyroidism Treated With Parathyroidectomy Versus Nonoperative Management. Ann Surg. 2023;278(2):e302-e8.

71. Lecoq AL, Livrozet M, Blanchard A, Kamenicky P. Drug-Related Hypercalcemia. Endocrinol Metab Clin North Am. 2021;50(4):743-52.

72. Griebeler ML, Kearns AE, Ryu E, Thapa P, Hathcock MA, Melton LJ, 3rd, et al. Thiazide-Associated Hypercalcemia: Incidence and Association With Primary Hyperparathyroidism Over Two Decades. J Clin Endocrinol Metab. 2016;101(3):1166-73.

73. Fommei E, Iervasi G. The role of thyroid hormone in blood pressure homeostasis: evidence from short-term hypothyroidism in humans. J Clin Endocrinol Metab. 2002;87(5):1996-2000.

74. Rivas AM, Pena C, Kopel J, Dennis JA, Nugent K. Hypertension and Hyperthyroidism: Association and Pathogenesis. Am J Med Sci. 2021;361(1):3-7.

75. Cappola AR, Desai AS, Medici M, Cooper LS, Egan D, Sopko G, et al. Thyroid and Cardiovascular Disease: Research Agenda for Enhancing Knowledge, Prevention, and Treatment. Circulation. 2019;139(25):2892-909.

76. Vidili G, Delitala A, Manetti R. Subclinical hyperthyroidism: the cardiovascular point of view. Eur Rev Med Pharmacol Sci. 2021;25(8):3264-71.

77. Stabouli S, Papakatsika S, Kotsis V. Hypothyroidism and hypertension. Expert Rev Cardiovasc Ther. 2010;8(11):1559-65.
